# A two-compartment model of synaptic computation and plasticity

**DOI:** 10.1186/s13041-020-00617-1

**Published:** 2020-05-20

**Authors:** Rudi Tong, Nigel J. Emptage, Zahid Padamsey

**Affiliations:** 1 Department of Pharmacology, University of Oxford, Mansfield Road, Oxford, OX1 3QT UK; 2 Current address: McGill University, Montreal Neurological Institute, 3801 University Street, Montreal, H3A 2B4 Canada; 3 Centre of Discovery Brain Sciences, University of Edinburgh, 9 George Square, Edinburgh, EH8 9XD UK

## Abstract

The synapse is typically viewed as a single compartment, which acts as a linear gain controller on incoming input. Traditional plasticity rules enable this gain control to be dynamically optimized by Hebbian activity. Whilst this view nicely captures postsynaptic function, it neglects the non-linear dynamics of presynaptic function. Here we present a two-compartment model of the synapse in which the presynaptic terminal first acts to filter presynaptic input before the postsynaptic terminal, acting as a gain controller, amplifies or depresses transmission. We argue that both compartments are equipped with distinct plasticity rules to enable them to optimally adapt synaptic transmission to the statistics of pre- and postsynaptic activity. Specifically, we focus on how presynaptic plasticity enables presynaptic filtering to be optimally tuned to only transmit information relevant for postsynaptic firing. We end by discussing the advantages of having a presynaptic filter and propose future work to explore presynaptic function and plasticity in vivo.

## Introduction

**Fig. 1:**
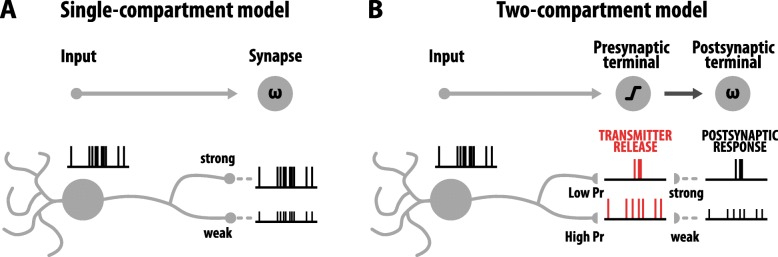
The two-compartment model of the synapse. **a** In the conventional single-compartment model, inputs are scaled linearly by the synapse, often characterised by a weighting factor w. **b** The addition of the presynaptic terminal results in a dynamic temporal filter prior to the postsynaptic gain control. This leads to a temporal decomposition of the presynaptic input spike train across presynaptic terminals along the axon. Importantly, this filter is dynamically regulated by ongoing neural activity to adjust to the statistics of pre- and postsynaptic activity

Historically, the synapse has been viewed as a single compartment in which synapse strength is represented by a simple multiplicative factor, a form of gain control that linearly scales incoming input. Synaptic plasticity enables this gain control to be dynamic, placed under the regulation of Hebbian activity. This view of the synapse lies at the heart of many successful experimental and computational models of neuronal and circuit function, and forms the basis of state-of-the-art machine learning algorithms [13, 37, 43]. Despite the success of this simple model of the synapse, these simplifications do not map well to the actual biology and physiology of the synapse, which consists of two distinct biological compartments: a pre- and a postsynaptic terminal. Whilst the idea of gain control nicely captures postsynaptic function, it does not capture the non-linear and stochastic nature of presynaptic transmitter release. Here, based on recent experimental evidence we propose a two-compartment model of the synapse, in which the presynaptic terminal first acts to filter presynaptic information before the postsynaptic terminal acts as a gain controller to amplify or depress its impact on the postsynaptic neurone. Importantly, we argue that each compartment must have unique plasticity rules to optimize its function, and discuss specifically, based on recent experimental evidence, how presynaptic plasticity optimally tunes presynaptic filtering to maximize efficient information transfer (Fig. 1). Our proposed model endows the synapse with a powerful means for optimally adapting synaptic transmission to the statistics of pre- and postsynaptic activity. We begin by discussing why the pre- and postsynaptic compartments should be viewed as mechanistically and functionally distinct contributors to synaptic transmission.

## Pre- and postsynaptic compartments are mechanistically and functionally distinct

For the purpose of simplicity, we will restrict our notion of pre- and postsynaptic strength to the probability of neurotransmitter release (Pr) and the current evoked by transmitter release (q), respectively. Notably, the *modus operandi* of the pre- and the postsynaptic terminal differ as presynaptic release is highly non-linear and stochastic, unlike the linear scaling implemented by the postsynaptic terminal. In general, the mechanistic and functional differences between pre- and postsynaptic terminals can be laid out as:

### 1. Pre- and postsynaptic terminals have access to distinct aspects of neural activity

Due to the stochastic nature of presynaptic release, the postsynaptic terminal experiences only the fraction of presynaptic action potentials that is translated into successful neurotransmitter release, while release failures are indistinguishable from the complete absence of action potentials. By contrast, the presynaptic terminal does not have direct access to the postsynaptic membrane potential and instead relies on the transmission of feedback signals via retrograde messengers.

### 2. Changes in pre- and postsynaptic strength have differential impact on the postsynaptic membrane response

Given the binomial theorem, Pr and q have a similar impact on the mean synaptic response (μ) but differentially impact response variability (σ^2^) [22].

1 $$ \mu =\mathit{\Pr}\times q\kern2em {\sigma}^2=\mathit{\Pr}\left( 1\hbox{-} \mathit{\Pr}\right){q}^2 $$

Moreover, whereas a change in q scales the amplitude of all postsynaptic potentials approximately linearly, the effect of Pr becomes only meaningful when responses are considered over multiple presynaptic spikes. The optimisation of presynaptic strength must therefore be made with respect to the temporal properties, such as the firing frequency, of the input.

### 3. Presynaptic strength is dependent on temporal patterns of neuronal activity

Neuronal firing patterns trigger short-term forms of plasticity, which predominantly impact pre-, rather than post-, synaptic function [56]. Short-term plasticity is sensitive to the temporal properties of the input and enables the presynaptic terminal to act as a temporal filter [1, 15, 32, 35, 58, 62–64]. The filtering properties of the presynaptic terminal are well-studied and depend on the basal Pr of the synapse. In particular, high Pr synapses typically exhibit short-term depression during high frequency bursts, which enable them to act as low-pass filters, preferentially releasing neurotransmitter in response to single spikes or low frequency spiking. By contrast, low Pr synapses typically exhibit short-term facilitation, which enable them to act as high-pass filters, preferentially releasing neurotransmitter in response to high frequency spiking [25, 26]. The impact of such synaptic non-linearities on network function has been extensively explored in silico using dynamic synapses employing phenomenological models of short-term plasticity [4, 40, 41, 44, 51, 55, 63]. For example, short-term presynaptic plasticity enables excitatory synapses to differentially transmit glutamate depending on relative changes in firing frequencies [2, 64], and to extract complex temporal patterns from presynaptic firing, such as precise spike-timing patterns [16, 40]. Despite much research into the non-linear dynamics of presynaptic function, little is known about how presynaptic terminals are optimally tuned to transmit information to their postsynaptic partners. Such tuning would require unique presynaptic learning rules that enable reliable transmitter release to be triggered by only patterns of presynaptic activity that are associated with postsynaptic spiking.

## The two-compartment model of the synapse

Given these initial observations, we suggest that the synapse should be viewed as two functionally distinct and independently regulated compartments, in the form of the pre- and the postsynaptic terminals (Fig. 1b). Importantly, each compartment is subject to a set of independent learning rules, which optimize their respective functions: postsynaptic plasticity enables the synapse to function as a dynamic gain control, whereas presynaptic plasticity enables the synapse to function as a dynamic temporal filter. This drastically increases the information processing capabilities of the synapse as presynaptic input can be first filtered before being amplified or depressed.

Although the pre- and the postsynaptic terminal have been previously viewed as a dynamic filter and gain controller [1], a key extension of our model is recognizing that the presynaptic terminal requires distinct learning rules from the postsynaptic terminal in order to optimally tune its function. In the following, we will briefly discuss the evidence that strongly suggests a molecular dissociation between pre- and postsynaptic plasticity mechanisms at the well-studied Schaffer-collateral synapses. We then introduce our recent discovery of a novel presynaptic plasticity rule at these synapses and discuss how it may optimally tune presynaptic filtering. We conclude with an outlook on the functional consequences of presynaptic filtering and propose necessary future experiments to explore its role and relevance in brain function in vivo.

## Pre- and postsynaptic plasticity are mechanistically and functionally distinct

**Fig. 2:**
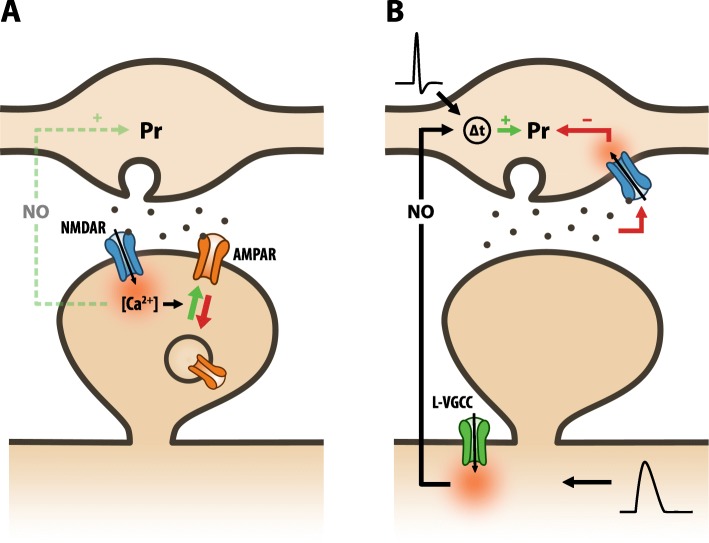
Pre- and postsynaptic plasticity are mechanistically distinct. **a** Conventional model of NMDAR-dependent synaptic plasticity at central synapses. Hebbian activity is sensed by postsynaptic NMDARs and translated into a postsynaptic influx of Ca^2+^. This leads to the exo- or endocytosis of AMPARs, which depends on the magnitude of Ca^2+^ influx. Additionally, NMDAR-dependent Ca^2+^ influx is conventionally thought to trigger the synthesis and release of retrograde signals such as nitric oxide (NO), which then modulates plasticity at the presynaptic terminal. **b** Novel model of presynaptic plasticity [53]. At the hippocampal Schaffer collateral-CA1 synapse, changes in Pr are driven by two parallel molecular mechanisms that are independent of postsynaptic NMDARs: 1) Presynaptic LTP, which is induced by Hebbian activity, involving the causal pairing of presynaptic action potentials and strong postsynaptic depolarisation. Postsynaptic depolarisation, in the form of dendritic spikes or back-propagating action potentials, driven by cooperative synaptic activity, triggers the synthesis and release of NO in dendritic branches [53]. At the presynaptic terminal, NO can increase Pr but only when presynaptic activity precedes its release. Such timing requirements are likely mediated by an as yet unidentified Hebbian coincidence detector in the presynaptic terminal. 2) Presynaptic LTD, which is triggered by glutamate release via the activation of presynaptic NMDARs. Accordingly, presynaptic LTP is preferentially induced at synapses releasing little or no glutamate during Hebbian activity

Long-term synaptic plasticity, such as long-term potentiation (LTP) and long-term depression (LTD), enables synapses to be optimally and dynamically tuned to the statistics of the environment. The most widely studied forms of Hebbian plasticity are NMDA receptor (NMDAR)-dependent LTP and LTD, in which NMDAR activity reports the extent of correlation between presynaptic glutamate release and postsynaptic activity. NMDAR-dependent forms of plasticity were traditionally thought to be mediated by both postsynaptic changes in AMPA receptor (AMPAR) number (q) and presynaptic changes in Pr (Fig. 2a, [7]). Whereas there is strong experimental evidence supporting a causal link between postsynaptic NMDAR Ca^2+^ influx and changes in AMPAR number, the link between postsynaptic NMDAR Ca^2+^ influx and changes in Pr has been far more tenuous. Indeed, a number of studies have now shown that presynaptic LTP can be obtained in NMDAR blockade in a manner dependent on Ca^2+^ influx from L-type voltage-gated Ca^2+^ channels (L-VGCCs) [5, 9, 53, 66]. Moreover, presynaptic LTD also appears to be independent of postsynaptic NMDARs and is instead driven by presynaptic NMDAR activity [3, 10, 57]. Thus, it appears that pre- and postsynaptic forms of plasticity are mechanistically distinct.

A mechanistic distinction in plasticity rules also suggests a functional distinction. It is well accepted that postsynaptic plasticity enables the synapse to adjust its gain in accordance with the correlation between presynaptic glutamate release and postsynaptic spiking [7, 43]. In this way, the postsynaptic terminal is optimised to promote the transmission of inputs that are associated with postsynaptic spiking. However, it is less clear what the functional role of presynaptic plasticity is, especially since changes in Pr, unlike changes in q, non-linearly impact synaptic transmission. Lowering Pr, for example, would preferentially depress inputs at low frequencies, but owing to short-term facilitation, would leave transmission at high frequencies little changed. Changes in q, by contrast, would similarly impact transmission across all input frequencies. Given the well described role of the presynaptic terminal as a frequency filter, a reasonable hypothesis is that presynaptic plasticity may optimise the presynaptic terminal to preferentially transmit presynaptic frequencies that are associated with strong postsynaptic spiking: if low frequency activity, or even single spikes, are associated with postsynaptic spiking, Pr should be set high to ensure efficient glutamate release during low frequency activity, whereas if high frequency activity is associated with postsynaptic spiking, Pr should be set to lower values to ensure glutamate is only released during high frequency activity. Consistent with this reasoning, we found that pairing high frequency bursts of presynaptic spikes with strong postsynaptic depolarisation at hippocampal synapses maintained Pr at low values, despite the presence of Hebbian activity and high levels of glutamate release. By contrast, pairing single presynaptic spikes with strong postsynaptic depolarization reliably increased Pr, despite lower levels of glutamate release [53]. Such findings diverge from predictions made by standard NMDAR-dependent models of postsynaptic plasticity, in which higher levels of glutamate released during Hebbian activity triggers larger increases in synaptic efficacy [29, 43, 45, 50]. These findings therefore prompted us to better investigate the mechanisms underlying presynaptic plasticity.

## New understandings of the mechanism of presynaptic plasticity

**Fig. 3:**
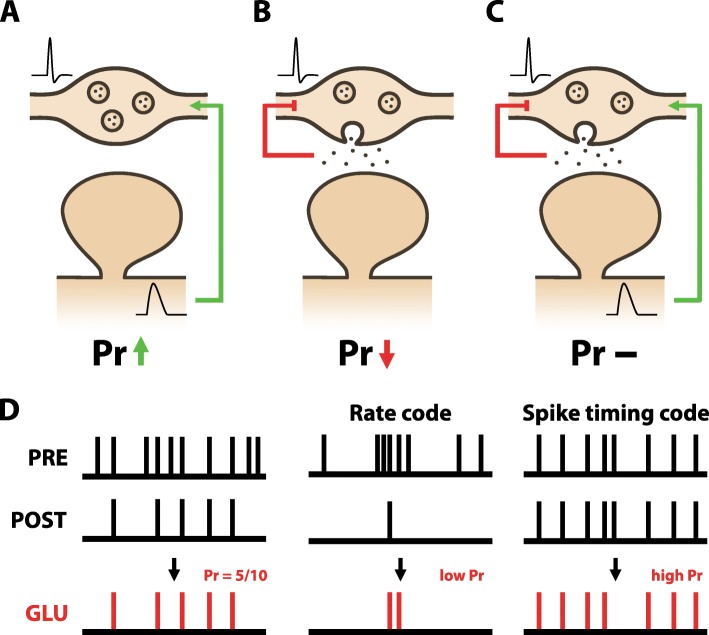
The presynaptic terminal adapts to the statistics of pre- and postsynaptic activity. The presynaptic learning rule can be understood as a minimisation of the prediction error between neurotransmitter release and postsynaptic spiking. **a** Hebbian activity, whereby postsynaptic spiking (in the form of somatic or dendritic spikes) is causally paired with presynaptic action potentials triggers an increase in Pr by a positive feedback signal (i.e. retrograde NO signalling). **b** Release of glutamate causes a decrease in Pr irrespective of postsynaptic spiking by a negative feedback signal (i.e. presynaptic NMDAR activation). **c** Positive and negative feedback signals work in parallel and cancel each other out when neurotransmitter release is followed by postsynaptic spiking. **d** This learning rule optimises Pr with respect to the conditional probability of postsynaptic spiking given prior presynaptic activity. At steady-state, each release event will be followed, on average, by a postsynaptic spiking event (Left). As a consequence, burst firing will generally result in low Pr (Middle), whereas synapses using spike-timing codes will tend towards high Pr (Right). Pr is therefore optimally tuned to preferentially transmit presynaptic input that is predictive of postsynaptic spiking

To better elucidate the mechanisms of presynaptic plasticity we manipulated the levels of glutamate signalling and Hebbian activity at hippocampal synapses and observed the resulting change in Pr using optical approaches [53]. We found that presynaptic LTP could be induced by Hebbian pairing of pre- and postsynaptic spiking in the complete absence of glutamate signalling. In particular, strong postsynaptic depolarisation, likely by driving dendritic spikes, activated L-VGCCs, which triggered retrograde release of nitric oxide (NO) from neuronal dendrites. Retrograde NO signalling was sufficient to trigger an increase in Pr provided that presynaptic terminals were active just prior to (7–10 ms), but not following, NO release (Fig. 2b). In this way, a presynaptic terminal could be potentiated without releasing glutamate, provided that its activity coincides with postsynaptic spiking, which in a physiological setting would be driven by glutamate release at other co-active synapses. When glutamate release did occur at synapses, we found that it decreased Pr by activating presynaptic NMDARs, and promoted presynaptic LTD. Such decreases were detected regardless of the accompanying levels of postsynaptic spiking (Fig. 2b). Overall, our findings show that net changes in Pr are driven by two parallel processes: 1) Hebbian activity, which increases Pr (Fig. 3a), and 2) glutamate release, which decreases Pr (Fig. 3b). Consequently, when both processes occur simultaneously, i.e. glutamate release is followed by postsynaptic spiking activity, Pr remains unchanged (Fig. 3c).

More generally speaking, our presynaptic learning rule describes a simple prediction error learning rule, akin to the Rescorla-Wagner model of classical conditioning, where the mismatch and the difference between the levels of glutamate released (Glu) and the levels of Hebbian activity (H) during synaptic activity amounts to a proportional change in Pr scaled by the constant η, which is known as the learning rate (Eq. 2).

2 $$ \varDelta Pr=\eta \left(H\hbox{-} Glu\right) $$

The above equation can be re-written in terms of probabilities of Hebbian activity (H) and glutamate release (Glu). Specifically, the probability of Hebbian activity (H) relates to the conditional probability of postsynaptic spiking given a presynaptic action potential [P(post|pre)]. Note that postsynaptic spiking can refer to either backpropagating action potentials or local dendritic spikes. Accordingly, high probabilities of Hebbian activity reflect a high likelihood that there will be a postsynaptic spike given a single presynaptic spike. For the purpose of simplicity, the probability of glutamate release (Glu) simply equates to basal Pr if we assume the absence of short-term dynamics that influence glutamate release. The model can be adjusted to take into account short-term dynamics by using available models of short-term plasticity which predict effective Pr for a given pattern of neuronal activity [32]. However, for the purposes of explanation we will consider the simpler formulation, in which case we can re-write the above equation as (Eq. 3):

3 $$ \varDelta Pr=\eta \left[P\left( post| pre\right)\hbox{-} \mathit{\Pr}\right] $$

Following this equation, changes in Pr will only occur when there is a mismatch between P(post|pre) and Pr during neuronal activity. Such changes will minimize this mismatch by bringing Pr closer in value to P(post|pre). Provided the statistics of the pre- and postsynaptic activity do not change, Pr will eventually reach a steady state at which ΔPr = 0. At this state, Pr will equate to P(post|pre); that is the probability of glutamate release will match the probability of Hebbian activity at the synapse.

## Functional consequences of the two-compartment model

### Presynaptic plasticity enables the presynaptic terminal to act as an optimal frequency filter

In contrast to traditional learning rules which are only driven by Hebbian activity, our new learning rule for presynaptic plasticity is additionally negatively regulated by glutamate release. This might explain previous reports showing that the degree and direction of presynaptic plasticity is negatively correlated with the initial Pr [28, 59]. As a consequence, presynaptic plasticity can optimize Pr with respect to the temporal structure of presynaptic firing. For instance, consider a pair of neurones for which burst firing in the presynaptic neurone is predictive of the occurrence of a postsynaptic spike. Mechanistically, for high Pr synapses, excessive glutamate will be released during the action potential burst and presynaptic LTD will predominate according to our proposed model of presynaptic plasticity, triggering a decrease in Pr. With continued activity, Pr will continue to decrease until it is sufficiently reduced such that the depressing effect of glutamate release is matched to the potentiating effects of NO signalling triggered by postsynaptic depolarization. Formally, at such steady-state (ΔPr = 0), Pr will match P(post|pre), and will be given by (Eq. 4):

4 $$ \mathit{\Pr}=P\left( post| pre\right)=\frac{N_{post}}{N_{pre}} $$

Here, for the purposes of illustration, we can simplify P(post|pre) to N_post_/N_pre_, where N_post_ is the number of postsynaptic spikes elicited by each presynaptic burst and N_pre_ is the average number of action potentials in a presynaptic burst (Fig. 3d). This simplification assumes that a single presynaptic spike can elict at most one postsynaptic spike (i.e. N_post_≤N_pre_) but allows us to demonstrate intuitively how our learning rule optimally sets Pr given the statistics of pre- and postsynaptic firing. For example, if high frequency bursts of presynaptic activity reliably predict single postsynaptic spikes, then Pr will tend to low values as N_post_/N_pre_ will be low. Given short-term facillitation at low Pr synapses, this change would ensure that only high frequency bursts of activity reliably release glutamate. Conversely, if single presynaptic spikes reliably predict single postsynaptic spikes such that N_post_/N_pre_ = 1, as might be the case for spike timing codes, then Pr will also equal 1 (Fig. 3d). Given short-term depression at high Pr synapses, this change would ensure that reliable glutamate release is mainly triggered by single spikes (or low frequency spiking).

**Fig. 4:**
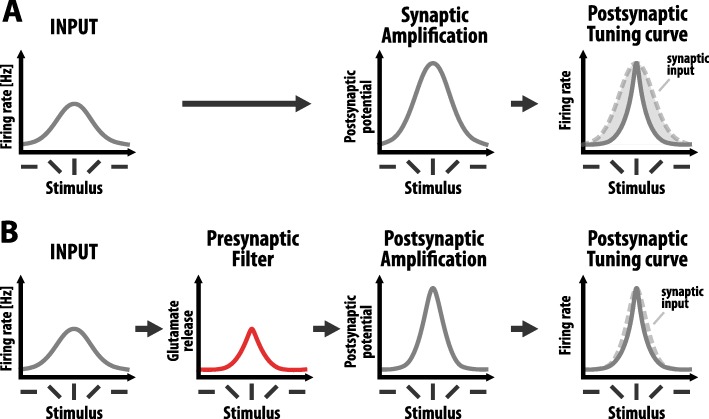
The presynaptic filter locally sharpens the tuning curve of the input neurone. **a** Example tuning curve of an orientation-selective neurone in the primary visual cortex. The single-compartment model leads to a postsynaptic amplification of the orientation tuning curve transmitted by the presynaptic neurone. This amplification uniformly impacts all presynaptic firing frequencies, which leads to the transmission of potentially irrelevant stimuli, for example when the tuning curve of the postsynaptic neurone is sharper (see mismatch between tuning curve of the input (dashed line) and tuning curve of the postsynaptic neurone). **b** In the two-compartment model, the same tuning curve is first sharpened due to the presynaptic frequency filter. For example, by using a low Pr synapse, high frequency presynaptic firing can be preferentially transmitted, enabling selective transmission of only relevant stimuli. The postsynaptic terminal can then selectively amplify this input. The two-compartment model can therefore optimize the signal-to-noise ratio of synaptic transmission

Our learning rule therefore enables Pr to be adjusted such that the bandwidth of presynaptic firing frequencies that are most informative or predictive of postsynaptic spiking will be transmitted most efficiently. This is relevant given that different frequencies of presynaptic firing are likely to convey different information [17]. The presynaptic terminal can therefore act as an optimal frequency filter and transmit only relevant information to the postsynaptic neurone. For example, Fig. 4a shows the hypothetical tuning curve of an orientation-selective neurone in the visual system. During synaptic transmission, the properties of the tuning curve are transmitted via the synapse from the presynaptic neurone to the postsynaptic neurone. If the postsynaptic neurone has sharper tuning than its presynaptic partner, then only a narrow band of presynaptic firing frequencies bears relevance for postsynaptic output. With the conventional, single-compartment synapse model, all presynaptic firing frequencies are transmitted via the synapse. Increasing (or decreasing) synaptic gain control can amplify (or depress) transmission, but would do so equally at all presynaptic firing frequencies, enhancing (or depressing) the transfer of both relevant and irrelevant information. By contrast, in the two-compartment model of the synapse, a presynaptic frequency filter could first be employed to filter out irrelevant presynaptic firing frequencies prior to postsynaptic amplification, thereby improving the signal-to-noise of synaptic transmission (Fig. 4b).

As a result of the presynaptic sharpening of tuning curves, presynaptic neurones can transmit a higher bandwidth of information, for example using a large range of firing frequencies, with only the relevant frequencies selected for at a given synapse. Similarly, a local synaptic filter allows the postsynaptic neurone to sample from a wider range of presynaptic inputs as each input will be dynamically tuned to maximally and optimally contribute to postsynaptic spiking. This, for example, alleviates connectivity constraints, in which neurones must selectively search for presynaptic partners with similarly tuned inputs.

Notably, our learning rule does not explicitly make assumptions about the nature of the postsynaptic spiking activity. This means that in the case of strongly electrically compartmentalised dendrites, presynaptic transmission will be optimised to local rather than global patterns of postsynaptic activity. For an axon that forms multiple contacts with a postsynaptic neurone, which is often the case for cortical neurones [38, 47, 61], this predicts that presynaptic terminals sharing the same dendrite will have more similar values of Pr than presynaptic terminals across different branches, which appears to be the case experimentally [12, 39].

### Beyond a simple presynaptic frequency filter

Our learning rule enables the presynaptic terminal to behave as an optimal frequency filter. However, the presynaptic terminal is capable of more complex forms of temporal filtering that can discriminate between different patterns of spiking, or between the onset and frequency of bursting activity [40, 41, 44, 64]. The optimization of such complex functions may likely require additional presynaptic plasticity rules than what we describe. Nonetheless, like frequency filtering, other forms of presynaptic filtering can be of considerable benefit for information processing in neuronal networks.

For example, both the onset and average frequency of burst firing can be differentially informative, but each requires distinct downstream mechanisms to be decoded. Conventionally, adjustments to cell intrinsic properties [8] or additional circuit mechanisms, such as inhibitory feedback [33], are needed to tune neurones to specific types of temporal information. However, with the addition of a local synaptic filter, each downstream neurone could independently adjust its synaptic filter to be sensitive to one or a combination of types of temporal information. In particular, high Pr synapses exhibiting short-term depression could be used to convey the timing of presynaptic bursts, invariant of burst frequency, whereas low Pr synapses exhibiting short-term facilitation could be used to convey information related to average burst frequency [48, 64]. Recently, transmission at the mossy fibre-CA3 synapse was reported to be sensitive to presynaptic spike number, independent of spiking frequency and timing [19]. Such functions, in principle, could also be mediated by presynaptic terminals that are tuned by presynaptic plasticity to have the appropriate short-term dynamics.

A presynaptic filter could also be used to normalize inputs. For example, a neurone might receive inputs from brain regions that differ in their average firing frequencies. In this case, inputs that have higher average spiking frequencies are also more likely to drive postsynaptic spiking. Synaptic integration at the postsynaptic neurone therefore becomes highly biased. A dynamic synaptic filter could, however, rescale the inputs to a similar frequency range. Moreover, a recent study reported that short-term plasticity of Schaffer collateral-CA1 synapses are altered along the length of CA1 dendrites in order to counteract electrotonic attenuation [27]; presynaptic filtering in such instance normalizes the contribution of synapses towards postsynaptic spiking independent of their position along the dendrite.

In summary, the two-compartment synapse model greatly increases the information processing capacity of neurones by allowing synaptic inputs to be locally filtered and adjusted. These filters can work complementary to those implemented on a circuit level, such as tight inhibitory feedback or organised connectivity and might alleviate some of their anatomical or metabolic constraints.

## Future work

Much remains to be understood about presynaptic filtering. Short-term plasticity and related synaptic non-linearities have been extensively studied in vitro, both on a phenomenological and molecular level [32, 56]. We believe that such a detailed in vitro characterisation of the synapse will continue to be invaluable for understanding the computational role of the synapse. These in vitro studies are, and will be, especially useful for setting bounds on what the synapse is capable of. Studies of synaptic plasticity will help clarify conditions driving synapse changes and the scale of these changes. For example, it will be crucial to determine the maximum temporal resolution of the synaptic filter. Can Pr be adjusted so that the resulting short-term dynamics of glutamate release is sensitive to arbitrary patterns of action potentials? Or can the presynaptic filter act only on the average frequency of spiking over a larger time window?

Of additional importance is better understanding the mechanisms regulating long- and short-term presynaptic function. Although we have elucidated the role of NO and glutamate release in regulating presynaptic function, other factors are also likely to be involved, including retrograde signalling by endocannabinoids [18], as well as glia-mediated release of glutamate and other gliotransmitters at the synapse [24]. How such factors impact presynaptic filtering remains to be elucidated. One possibility is that these factors might report aspects of postsynaptic activity on different spatial or temporal scales. To investigate this, a better understanding of the conditions that drive the release of different regulators of presynaptic function is needed. For example, we have shown that the release of NO requires the activation of L-VGCCs [53], which have high activation thresholds and fast inactivation kinetics, meaning that NO release preferentially encodes short periods of strong postsynaptic depolarisation.

Finally, of immense importance is the in vivo characterisation of synaptic function and plasticity rules, which is severely lacking, especially for the presynaptic terminal. Little is known about how ongoing basal activity, neuromodulatory tone, and interactions between excitatory and inhibitory activity, impact synaptic properties in vivo. Moreover, although it is established that both pre- and postsynaptic changes accompany learning in vivo [21, 36, 46], it remains unclear whether such learning rules are similar to those established in vitro*.* Indeed, the properties of one form of hippocampal synaptic plasticity observed in vivo during environmental exploration was recently reported to deviate substantially from in vitro findings [6]. Therefore, future studies need to focus on elucidating the properties of synaptic function and plasticity in vivo.

To better elucidate the function of the presynaptic terminal in vivo, we suggest the following guiding questions for future studies:

### What are the properties of stochastic presynaptic neurotransmitter release and short-term plasticity in vivo?

The lack of an in vivo characterisation of presynaptic properties can be largely attributed to technical difficulties of measuring Pr. Most techniques rely on the optical detection of neurotransmitter release events, which requires good optical access and probes that report transmitter release with high signal-to-noise ratios [52], previously not feasible for in vivo use. The recent development of novel, high-sensitivity Ca^2+^ and glutamate sensors should greatly facilitate the introduction of techniques such as optical quantal analysis into an in vivo setting [20, 23, 31, 34, 49, 54]. This paves the way for extensive characterisation of presynaptic properties at different connections and under different regimes of network activity.

### How are presynaptic properties regulated in vivo?

A consequence of the two-compartment synapse model is that the presynaptic filter is locally adjusted to optimize synaptic transmission between the pre- and the postsynaptic neurone. This predicts that presynaptic terminals along a common axon will differ in their release properties depending on the firing statistics of each associated postsynaptic neurone. This can be explored by imaging neurotransmitter release using glutamate sensors at boutons along identified axonal branches as these will experience the same presynaptic input but might differ in their pattern of release. Variations in transmitter release between boutons along the same axon should be explained by differences in the firing properties of the corresponding postsynaptic neurones. This can for example be assessed by recording or imaging activity from paired connections using Ca^2+^ or voltage-based sensors [14, 42, 54, 60].

Next, novel techniques for labelling recently potentiated synapses [30] can be combined with optical measurements of presynaptic function to study changes following learning, as well as pharmacological and genetic manipulations to study the underlying molecular mechanisms. These experiments would delineate similarities and differences in the induction, maintenance, and molecular underpinnings of pre- and postsynaptic plasticity, and can be compared with in vitro findings.

### How does the synaptic filter contribute to the input-output function of a neurone?

Lastly, the presynaptic filter needs to be understood in the context of a neurone’s input-output function. To do this, one has to compare qualitative differences between computational models of synaptic activity in vivo with and without presynaptic non-linearities. For example, improved circuit tracing techniques such as single cell-initiated retrograde tracing [65], in combination with genetically encoded reporters of activity, would make it possible to make simultaneous measurements of presynaptic action potentials, neurotransmitter release, and postsynaptic activity in vivo. These measurements can be used to construct models that map either 1) presynaptic action potentials to postsynaptic spiking or 2) glutamate release to postsynaptic spiking. If presynaptic filtering is significantly contributing to the generation of the input-output function, a qualitative difference between the models should be apparent. This can be used as a basis for further theoretical studies on the functional impact of the two-compartment synapse model on neural circuits.

## Conclusion

Viewing the synapse as more than a linear gain controller will help to better understand the role of the synapse in the emergence of complex network behaviour. Similar to the identification of local dendritic computation [11], the two-compartment model of the synapse will help to better assess the computational power of the neurone. This will lead to more precise models of synaptic integration and enable the formulation of more sophisticated hypotheses that can be tested experimentally. The rapidly expanding tool kit of novel techniques to interrogate synaptic function in vivo will encourage the translation of the expansive knowledge of the synapse in vitro into a physiological context.

## Data Availability

Not applicable (review).
